# Investigation of Renalase gene rs2576178 polymorphism in patients with coronary artery disease

**DOI:** 10.1042/BSR20180839

**Published:** 2018-09-14

**Authors:** Ning Hu, Jun Wang, Pengfei Hu, Zhongmei Li

**Affiliations:** 1 Department of Cardiology, Ningbo Municipal Hospital of Traditional Chinese Medicine, 819 Guang’an Road, Ningbo, Zhejiang, China; 2 Binjiang Clinic, Zhejiang Chinese Medical University, Hangzhou, Zhejiang, China; 3 Department of Cardiology, the Second Affiliated Hospital of Zhejiang Chinese Medical University, 318 Chaowang Road, Hangzhou, Zhejiang, China; 4 Department of Intensive Care Unit, Ningbo Municipal Hospital of Traditional Chinese Medicine, 819 Guang’an Road, Ningbo, Zhejiang, China

**Keywords:** coronary heart disease, molecular epidemiology, Renalase, rs2576178 polymorphism

## Abstract

Renalase gene rs2576178 polymorphism has been demonstrated to be a risk factor of ischemic stroke, essential hypertension, and end-stage renal disease, but the association Renalase with risk of coronary artery disease (CAD) has been less reported. Therefore, we detected Renalase rs2576178 polymorphism in 449 CAD patients and 507 healthy controls using matrix-assisted laser-desorption ionization (MALDI)/time of flight (TOF)-mass spectrometry (MS). It was found that GG genotype or G allele of rs2576178 polymorphism was associated with the risk of CAD. Stratified analysis indicated that Renalase polymorphism significantly increased the risk of CAD in females, smokers, and alcoholics. However, there was no significant association between different genotypes of rs2576178 polymorphism and clinical parameters. In summary, Renalase rs2576178 polymorphism is associated with increased risk of CAD, but this finding should be confirmed by larger studies with more diverse ethnic populations.

## Introduction

Coronary artery disease (CAD) is the main cause of morbidity and mortality in Western countries [1]. The lifetime risk of CAD at the age above 40 years is 49% for men and 32% for women [2]. Emerging evidence demonstrates that smoking, hypertension, diabetes, abdominal obesity, and lack of physical activity all contribute to the risk of CAD [3]. Parental CAD doubles the risk of CAD in the offspring, and sibling CAD increases the risk of incident CAD by 55% [4], indicating genetic risk factors play important roles in CAD development.

Renalase, a secreted amine oxidase synthesized in the kidney metabolizes circulating catecholamines and regulates blood pressure in both humans and animals [5]. Plasma Renalase level is markedly reduced in patients with chronic kidney disease or end-stage renal disease [6]. A Renalase knockout mouse model reveals that Renalase deficiency increases systolic and diastolic blood pressures [7]. Renalase protects renal injury and cardiac remodeling after subtotal nephrectomy via inhibiting inflammation, oxidative stress, and phosphorylation of extracellular signal-regulated kinases 1/2 [8]. A single-dose subcutaneous administration of recombinant Renalase significantly decreases blood pressure for more than 24 h in spontaneous hypertension stroke prone rats [9]. Hypertension is the most prevalent and significant modifiable risk factor of concomitant CAD [10]. To date, little is known about the role of Renalase gene in CAD development. One study shows the plasma Renalase level is significantly lower in CAD patients than the controls, indicating Renalase level is a factor of CAD [11].

The Renalase gene located on chromosome 10q23.31 has 16 exon counts. Though Renalase gene rs2576178 polymorphism is a susceptibility variant in various diseases, such as ischemic stroke [12], essential hypertension [13], and end-stage renal disease [14], little is known about its association with CAD susceptibility. Two studies exploring the association between Renalase polymorphism and risk of CAD present conflicting results [15,16]. Therefore, we performed this case–control study to examine the role of Renalase rs2576178 polymorphism in the risk of CAD in the Chinese population.

## Materials and methods

### Study population

In this case–control study on genetic association, a total of 449 CAD patients and 507 healthy controls were recruited from the Ningbo Municipal Hospital of Traditional Chinese Medicine or the Second Affiliated Hospital of Zhejiang Chinese Medical University between March 2013 and October 2017. CAD was diagnosed as at least 50% stenosis in one or more coronary angiographs. Subjects suffering from bacterial or viral infection, chronic inflammatory diseases, liver or kidney diseases, or any kind of cancer were excluded. The 507 controls without CAD at the time of sample collection were recruited from health examination. The healthy controls also had no known history of ischemic heart disease, endocrine, or metabolic disorders. Finally, medical and personal data from all participants were collected via a structure questionnaire. The present study was approved by the Clinical Research Ethics Committees of the two hospitals. Written informed consent was obtained from all the participants.

### Genotyping

Venous blood samples (2 ml each) were collected in tubes containing EDTA for genotyping analysis. Genomic DNA was extracted from peripheral blood using a QIAamp DNA blood mini-kit (Qiagen, Hilden, Germany) according to the manufacturer’s instructions. The concentration and purity of the extracted DNA were detected using NanoDrop at two optical density wavelengths 260 and 280 nm.

Rs2576178 is close to 5′-UTR of Renalase gene, which may affect the binding of transcription factors. This single nucleotide polymorphism (SNP) would cause the change from T to C nucleotides and has a minor allele frequency of 0.3442. Genotyping was done by matrix-assisted laser-desorption ionization (MALDI)/time of flight (TOF)-mass spectrometry (MS) using a MassARRAY system (Sequenom, San Diego, CA, U.S.A.). Complete genotyping reactions were spotted on a 384-well spectroCHIP (Sequenom) using a MassARRAY nanodispenser (Sequenom) and analyzed by MALDI-TOF-MS. Genotype calling was done in real time with MassARRAY RT 3.1 and analyzed using MassARRAY Typer 4.0 (both Sequenom). For quality control, repeated analyses were undertaken on 10% of randomly selected samples, and three positive and three negative controls were used. Accuracy of genotyping was determined by evaluating the genotype concordance between duplicate samples.

### Statistical analysis

Descriptive parameters were shown as mean ± S.D. or percentages. Between-group differences in means and proportions were analyzed via independent sample *t* tests and Pearson’s chi-square tests. Groups were compared via one-way ANOVA. The observed and expected genotype frequencies amongst controls were compared by testing the Hardy–Weinberg equilibrium (HWE) using a goodness-of-fit chi-squared test. Odds ratios (ORs) and 95% confidence intervals (CIs) were calculated to estimate the association between Renalase gene rs2576178 polymorphism and risk of CAD using logistic regression analyses. Power and sample size were calculated on the Power and Sample Size Program. The following parameters were used: type I error probability for a two-sided test (α); probability of exposure in controls (P0); number of patients (*n*); ratio of controls to patients (m); odd ratio of exposure in cases relative to controls (Ψ). All statistical analyses were performed on SAS package 9.1.3 (SAS Institute, Cary, NC, U.S.A.) at the significance level *P*<0.05.

## Results

### Characteristics of study subjects

The demographics and risk factors in CAD were listed in Table 1. The subjects were matched for age (*P*=0.786) and sex (*P*=0.795). The average body mass index (BMI) of 26.44 ± 3.96 kg/m^2^ in the patient group was higher than in the control group (*P*<0.001). Significant between-group difference was observed in high-density lipoprotein (HDL) (*P*<0.001), but not in total cholesterol (TC), low-density lipoprotein (LDL), smoking status, or alcoholism. No significant deviation from HWE was found for rs2576178 polymorphism in either cases or controls (*P*>0.05), suggesting the alleles were under equilibrium.

**Table 1:** Patient demographics and risk factors in CAD

Variable	Cases (*n*=449)	Controls (*n*=507)	*P*
Sex			0.786
Male	228 (50.77%)	253 (49.90%)	
Female	221 (49.22%)	254 (50.10%)	
Age (years)	55.70 ± 6.81	55.82 ± 7.04	0.795
BMI (kg/m^2^)	26.44 ± 3.96	23.07 ± 3.22	<0.001
TC (mmol/l)	4.78 ± 1.01	4.80 ± 1.00	0.790
HDL (mmol/l)	1.19 ± 0.33	1.38 ± 0.41	<0.001
LDL (mmol/l)	2.45 ± 0.49	2.47 ± 0.54	0.670
Smoking			0.092
No	185 (41.20%)	182 (35.90%)	
Yes	264 (58.80%)	325 (64.10%)	
Alcoholism			0.056
No	142 (31.63%)	132 (26.04%)	
Yes	307 (68.37%)	375 (73.96%)	

### Analysis of Renalase gene rs2576178 polymorphism

The distributions of Renalase rs2576178 polymorphism genotypes and alleles in the two groups were listed in Table 2. Our results revealed that Renalase rs2576178 polymorphism was significantly associated with increased risk of CAD (GG compared with AA, OR: 1.60, 95% CI: 1.07–2.39, *P*=0.022). We also confirmed this significant association in the recessive (*P*=0.031) and allelic (*P*=0.040) models.

**Table 2:** Logistic regression analysis of associations between rs2576178 polymorphism and risk of CAD

Genotype	Cases* (*n*=446)		Controls* (*n*=505)		OR (95% CI)	*P*
	*n*	%	*n*	%		
AG compared with AA	204/170	45.4/37.9	232/215	45.8/42.4	1.11 (0.84, 1.47)	0.450
GG compared with AA	72/170	16.0/37.9	58/215	11.4/42.4	**1.60 (1.07, 2.39)**	0.022
GG + AG compared with AA	276/170	61.4/37.9	290/215	57.2/42.4	1.21 (0.93, 1.57)	0.155
GG compared with AG + AA	72/374	16.0/83.3	58/447	11.4/88.2	**1.51 (1.04, 2.19)**	0.031
G compared with A	348/544	38.8/60.6	348/662	34.3/65.3	**1.22 (1.01, 1.47)**	0.040

Bold values are statistically significant (*P*<0.05).

* The genotyping was successful in 446 cases and 505 controls.

No significant association between genotypes of the Renalase rs2576178 polymorphism and clinical characteristics (age, BMI, TC, HDL, and LDL) was identified (Table 3). Stratified analyses showed Renalase rs2576178 polymorphism was significantly correlated with the increased risk of CAD in females, smokers, and alcoholics (Table 4). Power analysis indicated the present study had a power of 65.5% to detect the effect of rs2576178 polymorphism on CAD susceptibility, with OR of 1.11. The sample size of the present study reached 2209, which can increase the power to 0.8.

**Table 3:** The clinical and biochemical characteristics of Renalase gene rs2576178 polymorphism amongst two groups

	Patients (*n*=449)				Controls (*n*=507)			
	AA (*n*=170)	AG (*n*=204)	GG (*n*=72)	*P*	AA (*n*=215)	AG (*n*=232)	GG (*n*=58)	*P*
Age (years)	55.64 ± 6.86	55.32 ± 6.69	56.82 ± 6.96	0.273	55.88 ± 6.98	55.78 ± 7.17	55.48 ± 6.90	0.930
BMI (kg/m^2^)	25.96 ± 4.00	26.56 ± 3.74	27.00 ± 4.31	0.130	22.88 ± 3.16	23.10 ± 3.20	23.51 ± 3.34	0.388
TC (mmol/l)	4.80 ± 0.95	4.78 ± 1.08	4.74 ± 0.94	0.917	4.79 ± 0.93	4.81 ± 1.07	4.83 ± 0.93	0.959
HDL (mmol/l)	1.17 ± 0.34	1.23 ± 0.31	1.15 ± 0.35	0.129	1.39 ± 0.42	1.39 ± 0.39	1.29 ± 0.43	0.225
LDL (mmol/l)	2.40 ± 0.55	2.51 ± 0.46	2.42 ± 0.48	0.094	2.49 ± 0.53	2.45 ± 0.56	2.42 ± 0.53	0.620

**Table 4:** Stratified analyses between Renalase gene rs2576178 polymorphism and the risk of CAD

Variable	*rs2576178* (Case/control)			OR (95% CI); *P*			
	AA	AG	GG	AG compared with AA	GG compared with AA	AG + GG compared with AA	GG compared with AG + AA
Sex							
Male	96/105	96/121	35/27	0.87(0.59, 1.28); 0.471	1.47 (0.83, 2.62); 0.190	0.98 (0.68, 1.40); 0.890	1.59 (0.92, 2.73); 0.096
Female	74/110	108/111	37/31	1.45 (0.97, 2.15); 0.068	**1.77 (1.01, 3.12); 0.045**	**1.52 (1.04, 2.21); 0.029**	1.45 (0.87, 2.43); 0.159
Smoking							
No	69/67	87/92	28/22	0.92 (0.59, 1.43); 0.708	1.30 (0.67, 2.50); 0.442	0.99 (0.65, 1.51); 0.956	1.36 (0.74, 2.50); 0.322
Yes	101/148	117/140	44/36	1.23 (0.86–1.74); 0.260	**1.79 (1.08, 2.98); 0.025**	1.34 ( 0.96, 1.87); 0.083	**1.62 (1.01, 2.60); 0.048**
alcoholism							
No	57/63	65/55	20/14	1.31 (0.79, 2.17); 0.302	1.58 (0.73, 3.41); 0.246	1.36 (0.84, 2.20); 0.584	1.38 (0.67, 2.86); 0.384
Yes	113/152	139/177	52/44	1.06 (0.76, 1.47); 0.744	**1.63 (1.02, 2.61); 0.043**	1.17 (0.86, 1.59); 0.329	**1.58 (1.02, 2.44); 0.040**

Bold values are statistically significant (*P*<0.05).

## Discussion

This case–control study shows that Renalase gene rs2576178 polymorphism is associated with increased risk of CAD. Subgroup analyses of sex, smoking, and alcoholism uncover significant associations between rs2576178 polymorphism and CAD risk.

Renalase gene polymorphism is reportedly associated with cardiac hypertrophy, ventricular dysfunction, and inducible ischemia [17]. The association between Renalase gene rs2576178 polymorphism and CAD risk has been investigated [15,16]. Specifically, the frequency of allele A of rs2576178 polymorphism in hypertensive CAD patients is significantly higher than in both hypertensive patients and controls [16]. However, GG genotype of rs2576178 polymorphism is a protective factor of CAD compared with healthy controls [6]. Another study from Poland shows rs2576178 polymorphism is not associated with CAD compared with non-CAD [15]. It is noticeable that their controls are hemodialyzed patients without CAD, rather than healthy controls [18]. Our study shows Renalase gene rs2576178 polymorphism is a risk factor for CAD when compared with healthy controls and that the G allele or GG genotypes of this SNP carrier could increase the risk of CAD. Clearly, our findings are different from the other studies reporting association between rs2576178 polymorphism and decreased risk of CAD [16] or no significant association [15]. These discrepancies amongst studies may be attributed to five causes. First, geographical environments, sample sizes, and study designs are all different. Second, distributions of gene functional polymorphisms vary amongst races. Third, clinical heterogeneity may also be a cause, as our diagnostic criteria of CAD are different the other two studies. Fourth, CAD patients in their studies suffered from other diseases, such as hypertension and end-stage renal disease [15,16], which may affect the final results. Finally, we find significant associations between rs2576178 polymorphism and CAD risk in the subgroup analyses of sex, smoking, and alcoholism, which raises a possibility that sex, smoking, and alcoholism may interact with this SNP to increase the risk of CAD. To the best of our knowledge, this is the first study from China to uncover the risky role of Renalase gene rs2576178 polymorphism in CAD development.

As reported, increased plasma Renalase levels reduce systolic blood pressure (SBP) of resistant hypertensive patients [7]. SBP <120 mmHg is associated with adverse cardiovascular outcomes [19]. In addition, high Renalase levels and chronic kidney disease synergistically act to increase endothelin-1 levels, which is associated with endothelial dysfunction and vasoconstriction [20]. Thus, it is reasonable to assume that the elevated Renalase level is associated with the risk of CAD. Here our study shows that GG genotype of rs2576178 polymorphism confers susceptibility to CAD, indicating that GG genotype may increase Renalase levels and thereby contribute to increased risk of CAD.

Some limitations of this case–control study should be considered. First, the sample size was not large enough to obtain significant results. The study has a power of 65.5% to detect the effect of rs2576178 polymorphism on CAD susceptibility. Therefore, the limited sample size may result in false-positive or -negative results. Second, confounding factors such as sex and smoking may affect the results. Third, since this is a hospital-based study, selection bias may exist and the participants may not be representative of the general population. Fourth, no further subgroup analyses were conducted due to limited clinical information. Fifth, according to previous relevant literature, the tested polymorphisms may not provide a comprehensive view about the genetic variability of Renalase gene, which should be supplemented by further fine-mapping studies.

In conclusion, the GG genotype or G allele of rs2576178 polymorphism contributes to increased risk of CAD. This finding should be verified by studies in other populations.

## Abbreviations

BMI: body mass index
CAD: coronary artery disease
CI: confidence interval
HDL: high-density lipoprotein
HWE: Hardy–Weinberg equilibrium
LDL: low-density lipoprotein
OR: odds ratio
SBP: systolic blood pressure
SNP: single nucleotide polymorphism
TC: total cholesterol
